# Malignant epithelial cell marker–driven risk signature enables precise stratification in esophageal cancer

**DOI:** 10.3389/fimmu.2025.1610991

**Published:** 2025-05-27

**Authors:** Hao Zhang, Shizhao Cheng, Yijun Xu

**Affiliations:** ^1^ Tianjin Chest Hospital, Tianjin University, Tianjin, China; ^2^ Clinical School of Thoracic, Tianjin Medical University, Tianjin, China

**Keywords:** ESCC, scRNA-seq, epithelial cells, TME, keyword

## Abstract

**Background:**

Esophageal squamous cell carcinoma (ESCC) is a highly heterogeneous malignancy. Traditional clinical staging systems have limited value in prognostic evaluation and treatment guidance, often failing to capture the profound impact of intratumoral diversity on patient outcomes. Single-cell RNA sequencing (scRNA-seq) provides new perspectives on the cellular makeup and conditions in tumor tissues, with promising implications for functional classification and personalized therapeutic strategies in esophageal cancer.

**Methods:**

In this study, we integrated scRNA-seq data with bulk RNA-seq profiles from esophageal cancer tissues to construct a comprehensive cellular atlas, focusing on the transcriptional characteristics of epithelial cells. Malignant epithelial cells were identified based on copy number variation (CNV) features using inferCNV analysis. Their developmental states and regulatory mechanisms were further characterized via transcription factor activity inference (SCENIC) and pseudotime trajectory analysis (Monocle). Based on marker genes of malignant epithelial subpopulations, we developed a multi-gene risk scoring model using data from the TCGA and GEO (GSE53624) cohorts. The model’s predictive value for immune landscape, mutational features, and drug sensitivity was also evaluated. Additionally, qRT-PCR was conducted to quantify the expression levels of model genes in ESCC tissue samples, further evaluating their biological relevance. Functional roles of the key gene HMGB3 were validated *in vitro* through CCK-8 proliferation assays, Transwell invasion assays, and colony formation assays following gene knockdown in ESCC cell lines.

**Results:**

At the single-cell level, we identified ten major cell types and six distinct malignant epithelial subclusters, which exhibited pronounced heterogeneity in cell cycle states, transcriptional regulatory networks, and differentiation trajectories. High CNV scores and the enrichment of specific transcription factors (e.g., FOXC1, E2F1, RUNX1) suggested a proliferative and immune-evasive phenotype. A six-gene prognostic model (HMGB3, CHORDC1, CTSD, BTG2, MT1E, PHYHD1) showed strong predictive accuracy for overall survival in the TCGA and GSE53624 cohorts. Furthermore, the risk score showed a significant correlation with an immunosuppressive tumor microenvironment, increased tumor purity, and the activation of certain immune-related pathways. Analysis of drug sensitivity suggests that patients classified as low-risk could respond better to various targeted therapies and chemotherapeutic agents, underscoring their potential clinical relevance. Functional assays revealed that HMGB3 knockdown markedly suppressed ESCC cell proliferation, invasion, and colony formation, supporting its oncogenic role in esophageal cancer progression.

**Conclusion:**

This study systematically characterized epithelial cell heterogeneity in esophageal cancer at single-cell resolution and established a risk model based on malignant epithelial markers that effectively predicts prognosis, immune status, and potential drug response. Combined with experimental validation, our findings highlight the pivotal role of HMGB3 in ESCC progression and provide a novel theoretical and practical framework for functional tumor classification and individualized treatment strategies.

## 1 Introduction

Esophageal cancer (EC) is a highly aggressive digestive tract malignancy with poor clinical outcomes, accounting for more than 500,000 deaths annually worldwide and ranking among the top ten causes of cancer-related mortality (1, 2). Esophageal squamous cell carcinoma (ESCC), the predominant histological subtype in China and other East Asian countries, exhibits notable geographic distribution patterns (3). Over 70% of patients are diagnosed at advanced stages due to the disease’s insidious onset and absence of early symptoms, which greatly restricts treatment options and long-term survival. Although surgical techniques, chemoradiotherapy, and targeted therapies have advanced, the five-year survival rate for EC remains under 30% (4, 5).

In current clinical practice, the TNM staging system serves as the primary tool for risk assessment and treatment guidance in EC (6). However, growing evidence indicates that patients with the same clinical stage often exhibit substantial differences in prognosis, immune landscape, and therapeutic responsiveness. This highlights the limitations of traditional staging in capturing the underlying biological behavior and molecular heterogeneity of tumors. Such “clinical heterogeneity” represents a major obstacle to achieving truly personalized cancer treatment (7, 8). Simultaneously, the introduction of immune checkpoint inhibitors (ICIs) has transformed cancer treatment, driving critical research into biomarkers to identify EC patients who may respond to immunotherapy (9).

Single-cell RNA sequencing (scRNA-seq) has become a crucial method for analyzing the intricate cellular structure and diversity in tumor tissues. scRNA-seq offers high-resolution insights into individual cell types, cellular state transitions, and intercellular communication networks, unlike bulk RNA sequencing, which only provides averaged gene expression data across mixed cell populations (10, 11). This is particularly valuable in exploring the intricacies of the tumor microenvironment (TME) (12–14). Recent studies have shown that malignant epithelial cells comprise transcriptionally and functionally distinct subpopulations with diverse evolutionary trajectories (15). These subpopulations are closely associated with stemness, proliferative capacity, immune evasion, and potentially, therapeutic resistance and clinical outcomes. However, a comprehensive atlas of epithelial cell subpopulations in esophageal cancer remains lacking. Moreover, studies leveraging single-cell transcriptomic data to identify functional malignant subsets and construct prognostic models are still scarce.

Therefore, in this study, we systematically integrated scRNA-seq data from ESCC patients with large-scale bulk RNA-seq datasets to construct a detailed map of epithelial cell heterogeneity. Using multi-dimensional analyses, including CNV inference, pseudotime trajectory modeling, and transcription factor activity estimation, we identified potential malignant subclusters. Based on key marker genes derived from these subpopulations, we further developed a multi-gene risk model and evaluated its performance in predicting patient prognosis, immune microenvironment status, mutational landscape, and anticancer drug sensitivity. Our findings aim to provide novel cellular-level insights into ESCC heterogeneity and offer a theoretical and biological foundation for the refinement of precision therapeutic strategies

## 2 Methods

### 2.1 Data acquisition

Single-cell RNA sequencing data (PRJNA777911) were sourced from the National Center for Biotechnology Information’s Sequence Read Archive, as cited in a prior study (16). Transcriptomic data and clinical details of esophageal cancer patients were obtained from The Cancer Genome Atlas (TCGA).An independent external validation dataset was sourced from the Gene Expression Omnibus (GEO) database.

### 2.2 Single-cell atlas construction

Raw scRNA-seq data were processed in a Linux environment using CellRanger (17) for quality filtering, alignment, and generation of gene expression matrices. Downstream analyses were conducted with the Seurat (v4.0) R package (18–20). Seurat objects were created from multiple samples (min.cells = 5, min.features = 500), followed by data merging and calculation of mitochondrial gene proportion (pMT), hemoglobin gene proportion (pHB), and ribosomal gene proportion (pRP) for quality assessment. Cells were filtered based on the following criteria: 500 < nFeature_RNA < 9500, 1000 < nCount_RNA < 100000, pMT < 15%, and pHB < 3%. The data were normalized using the NormalizeData function and 3,000 highly variable genes were identified. Cell cycle effects were corrected using ScaleData with variables “S.Score” and “G2M.Score” after performing cell cycle scoring. Batch effects were removed via the Harmony algorithm (RunHarmony). Dimensionality reduction was performed using PCA, selecting the top 20 principal components for UMAP embedding. KNN graph construction (FindNeighbors) and clustering (FindClusters, resolution = 0.2) were used to identify cell subtypes. UMAP plots were generated to visualize sample distributions and clustering. Differential gene expression analysis was performed with FindAllMarkers (logFC > 0.5, only.pos = TRUE), and clusters were annotated based on published literature and public databases.

### 2.3 Cell-cell communication analysis

Intercellular communication in tumor and normal tissues was analyzed using the CellChat package (21). Expression matrices were extracted from the Seurat object, and ligand-receptor interactions were identified using the CellChatDB.human database. Communication probabilities (computeCommunProb) and pathway-level communication strengths (computeCommunProbPathway) were calculated, and intercellular communication networks were aggregated (aggregateNet). Differences between tumor and normal tissues were compared by signaling pathway activity (netAnalysis_computeCentrality) and visualized via network diagrams (netVisual_diffInteraction) and heatmaps (netVisual_heatmap). Bubble plots (netVisual_bubble) were used to display significantly altered signaling pathways.

### 2.4 Transcription factor regulatory network analysis

The SCENIC (22) workflow was applied to infer transcription factor (TF) activity. After gene filtering, co-expression networks were constructed using GENIE3, and regulons were identified (runSCENIC_1_coexNetwork2modules). Motif enrichment was assessed using the cisTarget database (runSCENIC_2_createRegulons), and TF activity was scored per cell (runSCENIC_3_scoreCells). UMAP visualization and AUC scores (getAUC) were used to assess TF activity across cell types. Key TFs were identified by comparing activity across clusters, followed by enrichment analysis. Heatmaps and violin plots were generated to display the expression patterns of core TFs.

### 2.5 Pseudotime trajectory analysis

Monocle 2 (23)was used to perform pseudotime analysis to infer the differentiation trajectories of epithelial cells. A CellDataSet was constructed using highly variable genes, followed by dimensionality reduction via DDRTree. A minimum spanning tree (MST) was used to establish developmental trajectories. Cell states and pseudotime distributions were visualized using plot_cell_trajectory. BEAM analysis was performed to identify branch-specific genes, and functional enrichment analyses were conducted to explore biological functions along the trajectory.

### 2.6 InferCNV analysis

To assess copy number variation (CNV) in epithelial cells, subsets were extracted from the Seurat object, normalized, and batch-corrected using Harmony. The inferCNV package (24) was applied with gene ordering based on the hg19 genome. Normal cells were used as reference, with a cutoff of 0.1 and denoising enabled. CNV heatmaps were generated, and malignant cells were identified by calculating CNV scores and Pearson correlation with the top 5% CNV-high cells (cor.estimate > 0.2). Malignant and normal epithelial cells were visualized on the UMAP plot.

### 2.7 Association of malignant epithelial subtypes with prognosis

Malignant epithelial cells identified by inferCNV were analyzed for marker gene expression using FindAllMarkers. Subtype scores were calculated in the TCGA cohort using ssGSEA. Kaplan-Meier analysis was used to assess associations between subtype scores and survival. Patients were stratified into high- and low-risk groups based on optimal cut-off values, and survival curves were plotted accordingly.

### 2.8 Feature gene selection and prognostic model construction

Marker genes from prognostically relevant epithelial subtypes were overlapped with differentially expressed genes from TCGA and GSE53624 to identify candidate genes. Univariate Cox regression was performed to identify prognostic genes (p < 0.05), visualized by forest plots. LASSO regression was then applied to avoid overfitting and select key variables, using 10-fold cross-validation to determine the optimal penalty parameter. The minimum mean cross-validation error corresponded to an optimal lambda value of 0.0529. Genes with nonzero coefficients under this lambda were selected for subsequent modeling. Multivariate Cox regression was subsequently conducted to construct the final prognostic model, with the Akaike Information Criterion (AIC) employed for further optimization. Risk scores were calculated for each patient, and individuals were stratified into high- and low-risk groups based on the median risk score. The TCGA cohort served as the training set, and GSE53624 was used as the independent validation cohort.

### 2.9 From cox regression to nomogram construction

Univariate and multivariate Cox regression analyses (25) were performed to evaluate the prognostic significance of clinical variables (Stage, Gender, Age) and risk scores. A nomogram was constructed to predict 1-, 3-, and 5-year survival probabilities based on independent prognostic factors. Calibration curves assessed model performance, and decision curve analysis (DCA) evaluated clinical utility across different threshold probabilities.

### 2.10 Pathway analysis associated with risk stratification

Gene Set Variation Analysis (GSVA) (26) and Gene Set Enrichment Analysis (GSEA) (27) were performed to compare pathway activities between risk groups. Hallmark gene sets from MSigDB (28)were used to calculate GSVA scores, and limma was used to identify significantly enriched pathways. GSEA was conducted using GO gene sets, and enrichment plots were generated. ssGSEA was applied to immune-related and cancer-immunity cycle pathways, and Pearson correlation analyses were performed between pathway activity and risk scores.

### 2.11 Tumor microenvironment infiltration analysis

ssGSEA (29) was used to calculate immune function scores and visualize group differences using heatmaps (ComplexHeatmap) and radar plots (ggradar). ESTIMATE (30) scores (ImmuneScore, StromalScore, TumorPurity) were calculated and correlated with risk scores using Pearson correlation and scatter plots to investigate associations with tumor progression.

### 2.12 Mutation landscape based on risk scores

Somatic mutation data from the TCGA-ESCA cohort were obtained via TCGAbiolinks (31). Samples were filtered and processed using maftools. Oncoplots illustrated the mutational distribution between high- and low-risk groups. Tumor mutational burden (TMB), mutation types, and co-occurrence or mutual exclusivity between high-frequency mutated genes were analyzed.

### 2.13 Individualized drug sensitivity prediction

Drug sensitivity was predicted using the oncoPredict (32) package based on transcriptome data from TCGA-ESCA. Gene expression data were preprocessed and matched to the GDSC2 reference dataset. Sensitivity scores were calculated for each patient. Wilcoxon tests were used to compare high- and low-risk groups, and significantly different drugs were visualized via boxplots, violin plots, and scatter plots.

### 2.14 RNA extraction and qRT-PCR validation

Total RNA was extracted with TRIzol reagent (Invitrogen) and reverse-transcribed using the PrimeScript RT kit (Takara). qRT-PCR was performed with SYBR Green Master Mix on an ABI 7500 system. GAPDH was used as an internal control, and relative expression was calculated using the 2^–ΔΔCt method.

### 2.15 Cell culture and transfection

Human ESCC cell lines (KYSE-150 and KYSE-410) were cultured in RPMI-1640 medium supplemented with 10% FBS and antibiotics. Gene knockdown was achieved using siRNAs (Obio Technology) transfected with Lipofectamine™ 3000 (Invitrogen) according to the manufacturer’s instructions. After 48 hours, cells were collected for downstream analyses. Transfection efficiency was confirmed by qRT-PCR and Western blot.

### 2.16 CCK-8 cell proliferation assay

KYSE-150 and KYSE-410 cells were seeded in 96-well plates (2,000–3,000 cells/well) post-transfection. Cell proliferation was assessed at 0 h, 24 h, 48 h, and 72 h using the CCK-8 assay (Dojindo, Japan), and absorbance was measured at 450 nm. Each group included five replicates.

### 2.17 Colony formation assay

Cells were seeded in 6-well plates (500–1,000 cells/well) and cultured for 10–14 days. Colonies were fixed with 4%paraformaldehyde and stained with 0.1% crystal violet. Colonies were photographed and counted under a microscope (33).

### 2.18 Transwell migration and invasion assay

Migration and invasion assays were performed using Transwell chambers with 8 μm pores. For invasion assays, upper chambers were pre-coated with Matrigel. After 24–48 h incubation, non-migrated cells were removed, and migrated cells were fixed, stained, and counted in five random fields.

### 2.19 Statistical analysis

All statistical analyses were conducted using R (v4.1.2) and GraphPad Prism 9. Data were presented as mean ± standard deviation (SD). Group comparisons were performed using two-tailed t-tests or one-way ANOVA as appropriate. Kaplan–Meier survival curves and log-rank tests assessed survival differences. Cox regression identified prognostic factors, and hazard ratios (HRs) with 95% confidence intervals (CIs) were reported. Nomograms and calibration curves were generated using the rms package (34), and decision curve analysis (DCA) (35)was used to evaluate clinical utility. A p-value < 0.05 was considered statistically significant.

## 3 Results

### 3.1 Construction of the single-cell atlas

**Figure 1:**
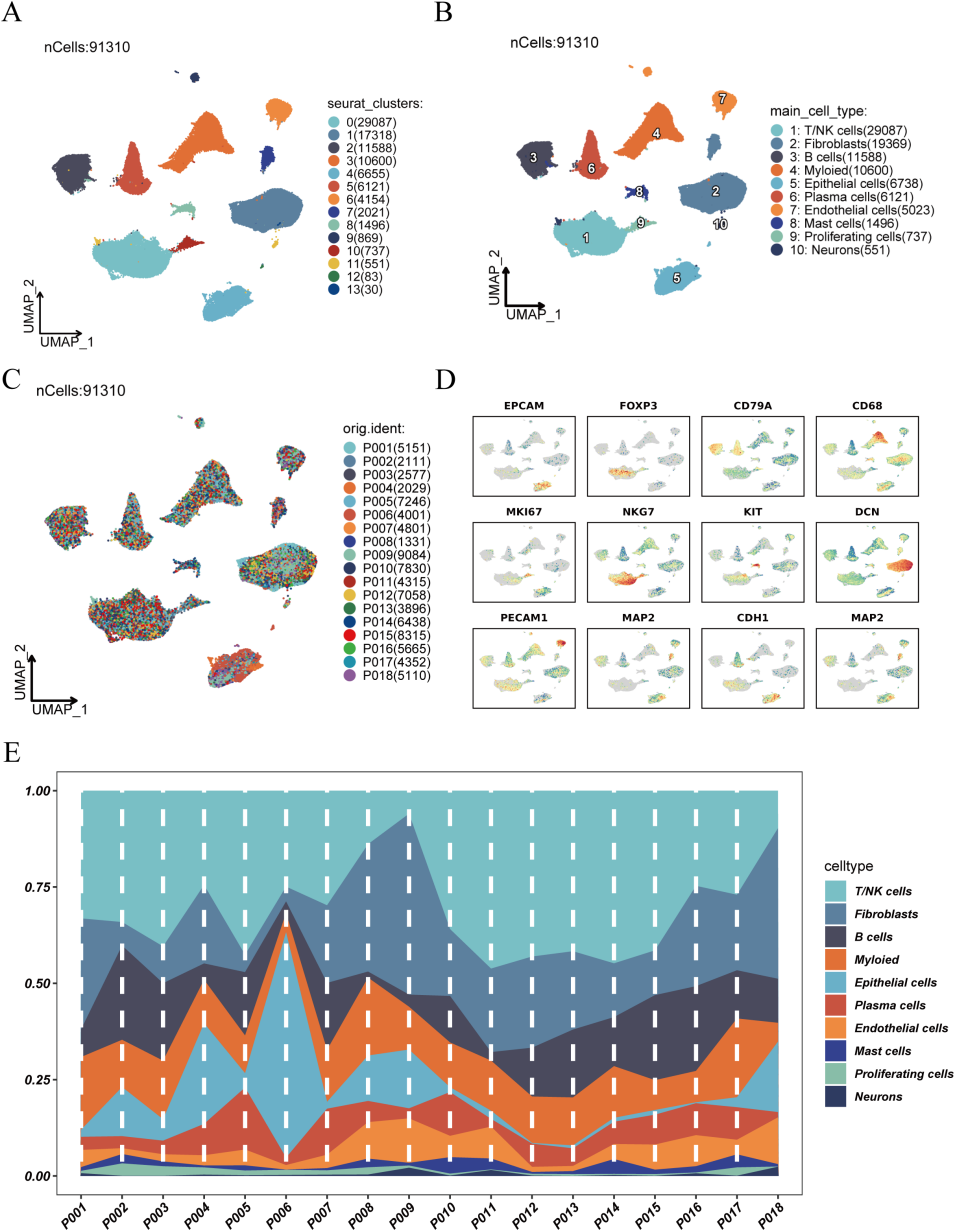
Clustering, annotation, and cell composition analysis of single-cell RNA sequencing data. **(A)** UMAP dimensionality reduction showing clustering results of scRNA-seq data, identifying 14 cell clusters. **(B)** Annotation of cell clusters based on known marker genes, identifying 10 major cell types: T/NK cells, fibroblasts, B cells, myeloid cells, epithelial cells, plasma cells, endothelial cells, mast cells, proliferating cells, and neurons. **(C)** Distribution of cells from different samples in UMAP space. **(D)** Expression patterns of representative marker genes across cell clusters, aiding in cell type identification. **(E)** Distribution of cell type abundance across individual patient samples, revealing inter-patient heterogeneity in cell composition.

After quality control and batch effect correction (**Supplementary Figure 1**), a total of 91,310 high-quality single cells were obtained and clustered using UMAP dimensionality reduction, resulting in the identification of 14 distinct cell clusters (**Figure 1A**). Based on known marker genes, we annotated 10 major cell types (**Figure 1B**), among which T/NK cells were the most abundant, followed by fibroblasts and B cells. The origin of each cluster was visualized by sample (**Figure 1C**), demonstrating that all clusters included cells from multiple patients, indicating successful data integration without significant batch effects. Cell type–specific marker gene expression analysis (**Figure 1D**) showed expected expression patterns across cell types, such as enrichment of EPCAM in epithelial cells, CD79A in B cells, and CD68 in myeloid cells. Finally, we profiled cell type composition across individual patient samples (**Figure 1E**), revealing substantial inter-patient heterogeneity. T/NK cells were dominant in most samples, while other cell types varied in abundance among patients.

### 3.2 Cell–cell communication analysis

**Figure 2:**
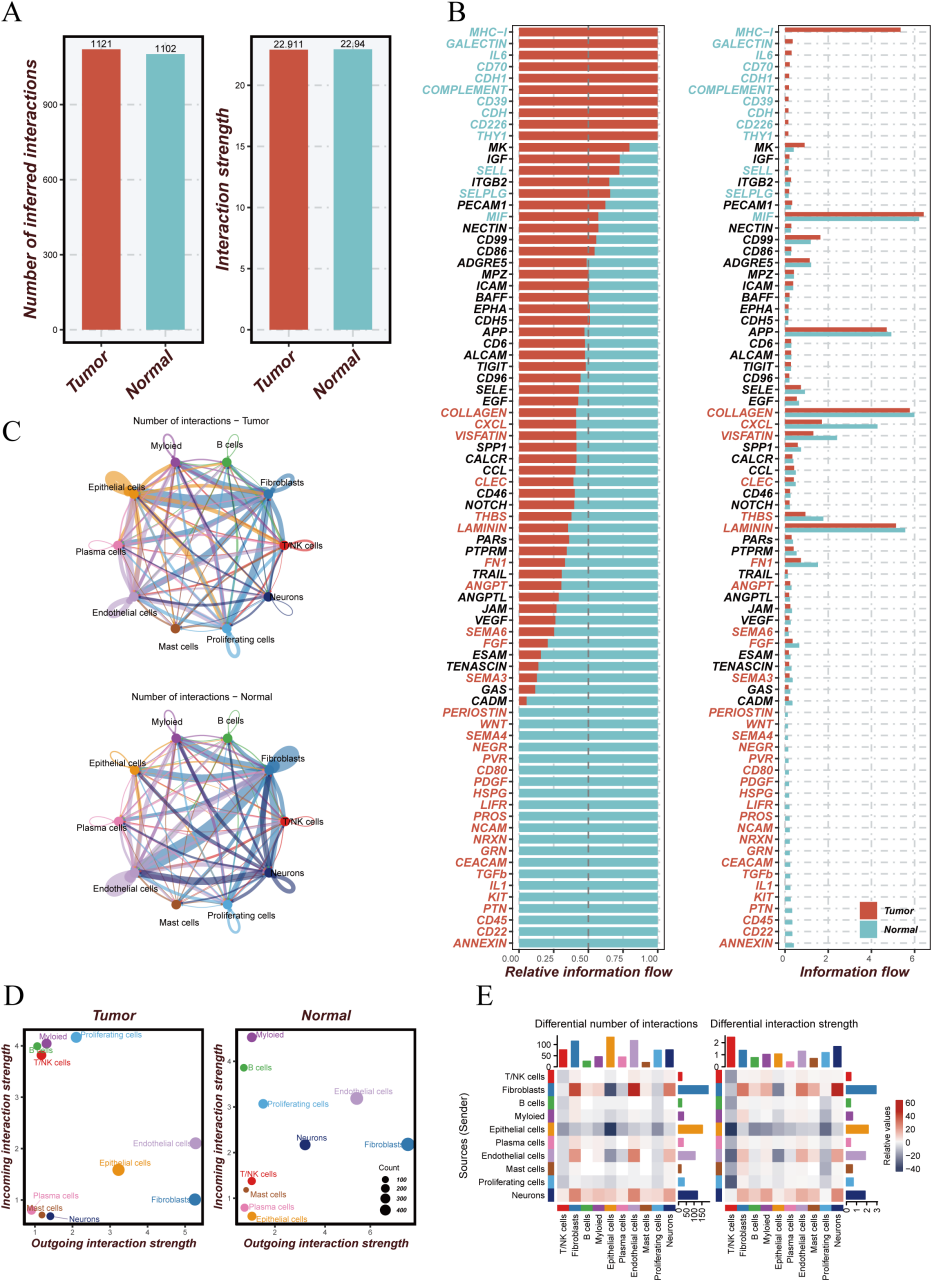
Analysis of cell–cell communication in tumor versus normal tissue microenvironments. **(A)** Comparison of the number and strength of intercellular communications between tumor and normal tissues. The left panel shows more interactions in tumor (red) than in normal (blue) tissue; the right panel shows greater interaction strength in tumors. **(B)** Information flow analysis of signaling pathways, displaying relative signal flow intensities in tumor (red) and normal (blue) tissues. **(C)** Cell–cell communication networks between different cell types in tumor (top) and normal (bottom) tissues; edge color and thickness indicate interaction strength. **(D)** Interaction strength of different cell types; x-axis represents outgoing interactions, y-axis incoming interactions, and bubble size indicates the number of interactions. **(E)** Differential interaction analysis showing changes in the number and strength of interactions across cell types between tumor and normal tissues; color intensity represents degree of change.

To investigate changes in intercellular communication within the tumor microenvironment, we conducted a comparative analysis between tumor and normal tissues. The number (1121 vs. 968) and overall strength (22,911 vs. 23,012) of cell–cell interactions were both elevated in tumor tissues compared to normal tissues (**Figure 2A**), suggesting more active communication in the tumor microenvironment. Signaling flow analysis revealed that pathways such as MHC-I, GALECTIN, and CD70 were more active in tumors, while matrix-related signals like COLLAGEN and LAMININ were more enriched in normal tissues (**Figure 2B**). Network visualization (**Figure 2C**) illustrated complex interactions among endothelial, epithelial, and fibroblast cells in tumors. Communication strength analysis (**Figure 2D**) indicated that T/NK cells in tumor tissues exhibited stronger inward interactions, whereas those in normal tissues had more balanced communication. Further comparison of communication networks across cell types (**Figure 2E**) showed enhanced interactions between fibroblasts and other cell types in tumors, while fibroblast–endothelial cell interactions were more prominent in normal tissues. These findings highlight dynamic alterations in intercellular signaling within the tumor microenvironment, offering insights into their role in cancer progression.

### 3.3 Re-clustering and copy number variation analysis of epithelial cells

**Figure 3:**
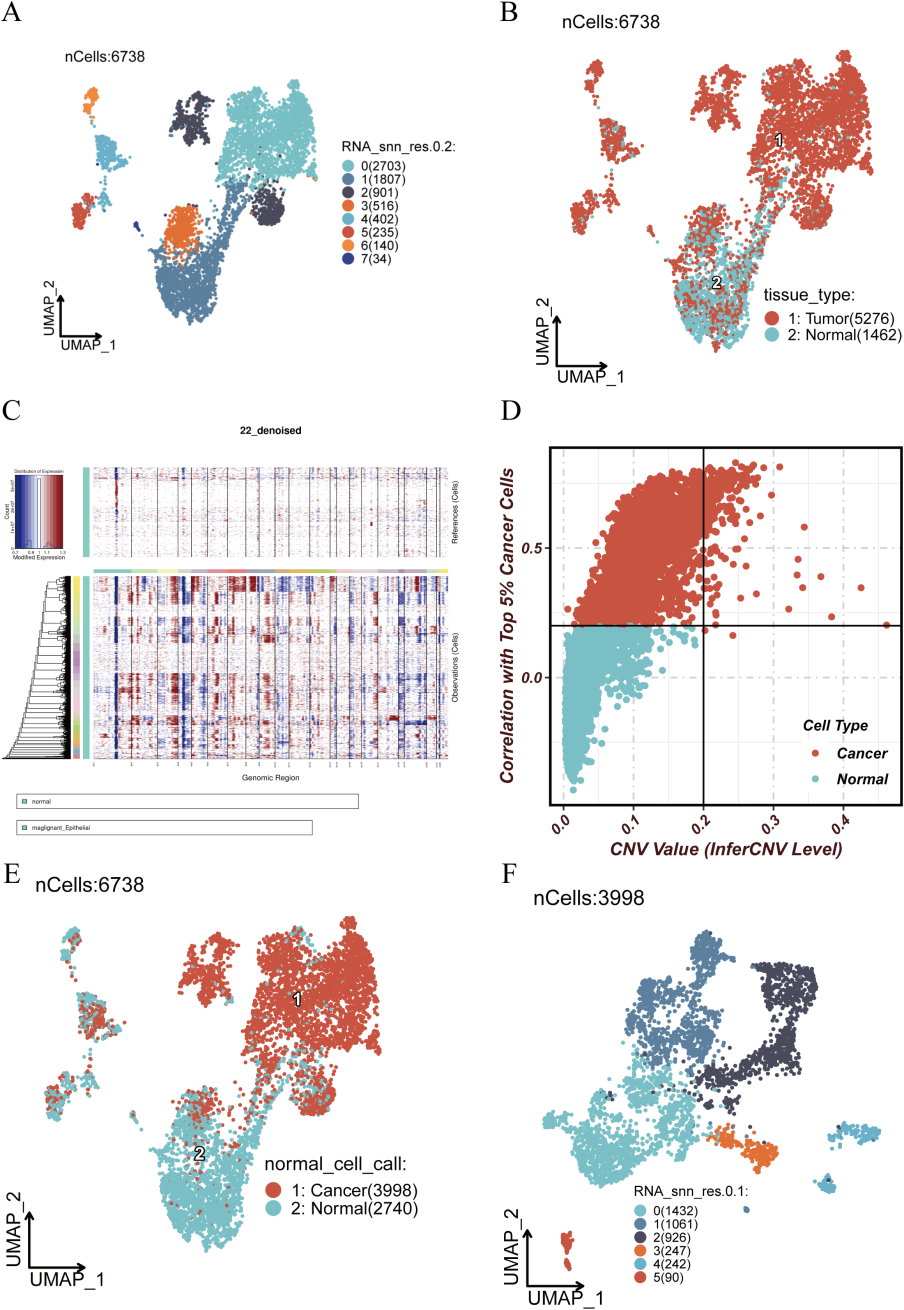
Re-clustering and inferCNV analysis of epithelial cells. **(A)** UMAP visualization of re-clustered epithelial cells showing distinct subpopulations. **(B)** Spatial distribution of epithelial cells stratified by tissue origin (tumor vs. normal). **(C)** inferCNV analysis showing copy number variations (CNVs) across genomic regions; red indicates amplifications, blue indicates deletions. **(D)** Correlation between CNV levels and malignant status; x-axis shows inferred CNV score, y-axis shows Pearson correlation with top 5% malignant cells. **(E)** Classification of cells into malignant (red) and normal (blue) based on inferCNV results. **(F)** Re-clustering of malignant epithelial cells to explore their internal heterogeneity.

To further explore epithelial cell dynamics in tumor tissues, we isolated epithelial cells and performed re-clustering using UMAP, identifying multiple epithelial subpopulations (**Figure 3A**). Stratification by tissue origin (tumor vs. normal) revealed distinct spatial distributions (**Figure 3B**). CNV analysis using inferCNV (**Figure 3C**) demonstrated prominent genomic alterations in tumor epithelial cells, including amplification and deletion events in specific chromosomal regions. Correlation analysis (**Figure 3D**) indicated that certain epithelial subclusters exhibited high CNV levels and were strongly associated with malignant phenotypes, while others showed low CNV and resembled normal cells. Based on inferCNV results, we classified epithelial cells as malignant or normal (**Figure 3E**), identifying 3,998 malignant and 2,740 normal cells. Malignant cells were further re-clustered (**Figure 3F**) to dissect their internal heterogeneity and provide a basis for downstream functional analysis.

### 3.4 Transcriptional regulation and pseudotime trajectory analysis of malignant epithelial cells

**Figure 4:**
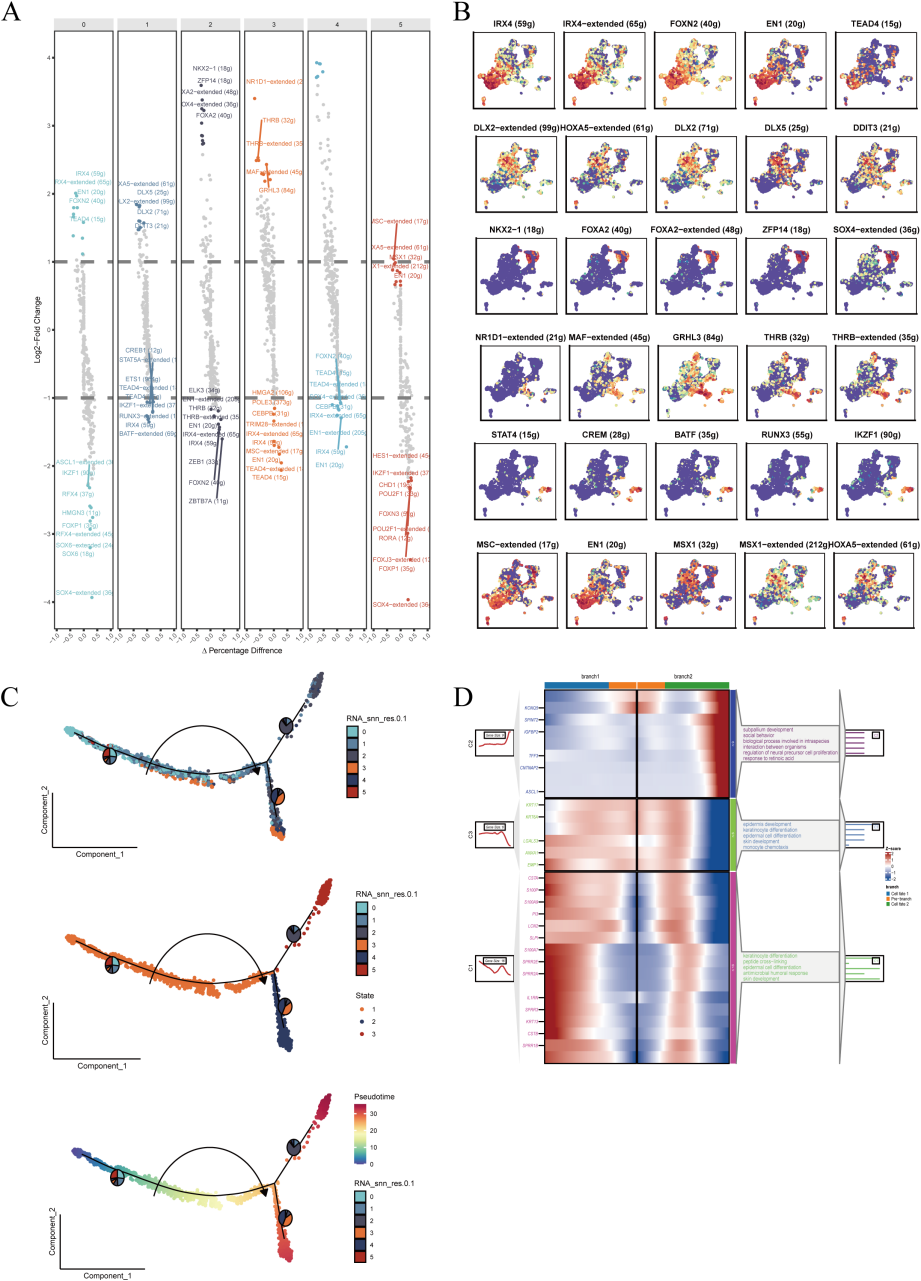
Transcriptional regulation and pseudotime trajectory analysis of malignant epithelial cells. **(A)** Differentially expressed transcription factors (TFs) enriched in each subpopulation, ranked by expression and significance. **(B)** Top five TFs in each cluster visualized in UMAP space to assess subtype-specific expression patterns. **(C)** Pseudotime trajectory reconstruction showing cellular progression and potential differentiation branches; color depth indicates cell state evolution. **(D)** Enrichment analysis of genes at key branching points in pseudotime, identifying related biological processes and signaling pathways.

To investigate the transcriptional regulatory landscape of malignant epithelial cells, we conducted differential expression analysis of transcription factors (TFs) across subclusters. Distinct TF expression patterns were observed in different malignant subpopulations (**Figure 4A**), suggesting potential roles in defining cellular function and fate. Key TFs highly expressed in specific clusters (**Figure 4B**) showed unique spatial distributions on the UMAP, indicative of divergent differentiation trajectories. For example, FOXC1 and E2F1 were enriched in certain clusters and may be linked to proliferation and cell cycle regulation, while RUNX1 and GATA3 were upregulated in others, potentially associated with lineage commitment. TF families such as DLX and IRX, known regulators of epithelial morphology and differentiation, were also highly expressed in select clusters.

Subsequently, we applied pseudotime trajectory analysis to reconstruct developmental progression within malignant epithelial cells (**Figure 4C**). Cells followed a continuous trajectory with multiple branches, reflecting diverse transitional states. Enrichment analysis of branch-specific genes (**Figure 4D**) revealed significant associations with biological processes such as proliferation and metabolic regulation, indicating dynamic transcriptional shifts during tumor progression. These findings suggest that malignant epithelial subtypes may represent distinct developmental or differentiation states shaped by specific TF networks.

### 3.5 Survival analysis of malignant epithelial subpopulations

To assess the clinical relevance of malignant epithelial subclusters, we calculated the enrichment of each subpopulation in bulk RNA-seq samples using single-sample gene set enrichment analysis (ssGSEA). Signature gene sets for each subcluster were derived from the single-cell data and applied to TCGA samples. Patients were stratified into high- and low-expression groups using optimal cutoff values, and Kaplan–Meier survival analysis was performed.

The results (**Supplementary Figure 2**) showed that high expression of Cluster 1 (p = 0.014), Cluster 3 (p = 0.032), and Cluster 4 (p = 0.0016) was significantly associated with worse overall survival. In contrast, Clusters 0 (p = 0.09), 2 (p = 0.13), and 5 (p = 0.14) showed no significant prognostic impact, suggesting inter-subtype heterogeneity in clinical outcomes and indicating that certain malignant epithelial subpopulations may serve as prognostic biomarkers.

### 3.6 Construction of the prognostic model

**Figure 5:**
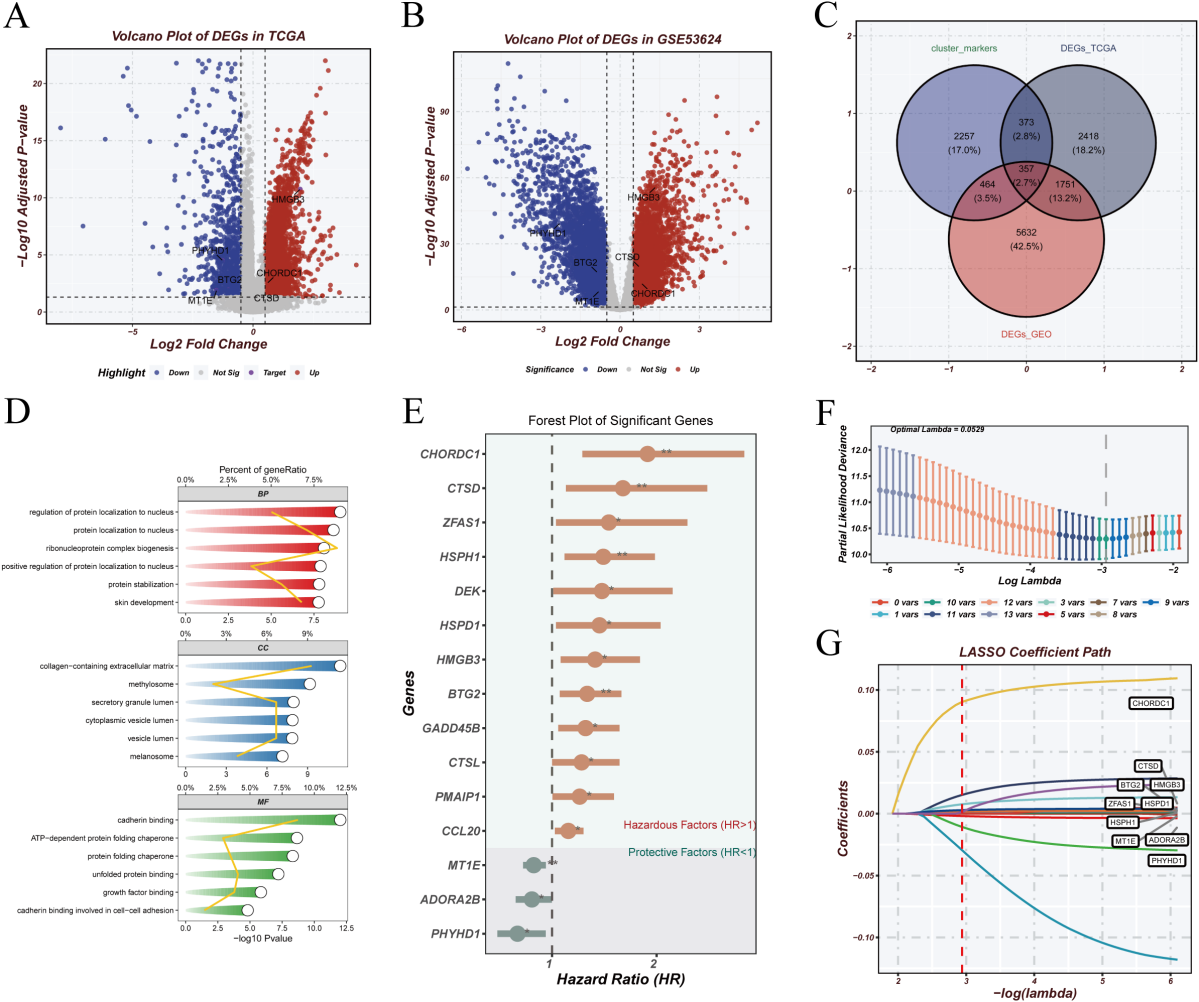
Model construction. **(A, B)** Volcano plots showing differentially expressed genes (DEGs) in TCGA and GSE53624 cohorts, with upregulated genes in red and downregulated genes in blue. **(C)** Venn diagram showing overlap between DEGs from TCGA, GEO, and marker genes from prognostic malignant epithelial clusters. **(D)** GO enrichment analysis revealing biological functions associated with the intersecting genes. **(E)** Univariate Cox regression analysis identifying survival-related genes; forest plot shows hazard ratios (HRs). **(F, G)** LASSO regression identifying six key genes; x-axis represents log(lambda), y-axis shows regression coefficients.

To develop a risk model based on malignant epithelial features, we first identified differentially expressed genes (DEGs) in the TCGA (**Figure 5A**) and GSE53624 (**Figure 5B**) cohorts. Overlapping the DEGs with marker genes of prognostic epithelial clusters (**Figure 5C**) yielded candidate genes. Gene Ontology (GO) enrichment analysis (**Figure 5D**) revealed significant enrichment in biological processes such as protein localization, extracellular matrix structure, cell adhesion, and signaling transduction. Univariate Cox regression identified survival-related genes (**Figure 5E**), followed by LASSO regression (**Figures 5F-G**) for dimensionality reduction and variable selection. Six genes were retained for model construction: HMGB3, CHORDC1, CTSD, BTG2, MT1E, and PHYHD1, of which the first four were risk factors (HR > 1) and the latter two were protective (HR < 1). A multivariate Cox regression model was constructed using these genes. The risk score was calculated as: RiskScore = (0.0276 × HMGB3) + (0.1461 × CHORDC1) + (0.0151 × CTSD) + (0.0046 × BTG2) − (0.0042 × MT1E) − (0.1389 × PHYHD1). This model was used to stratify patients into high- and low-risk groups for prognostic evaluation.

### 3.7 Model validation and prognostic performance assessment

**Figure 6:**
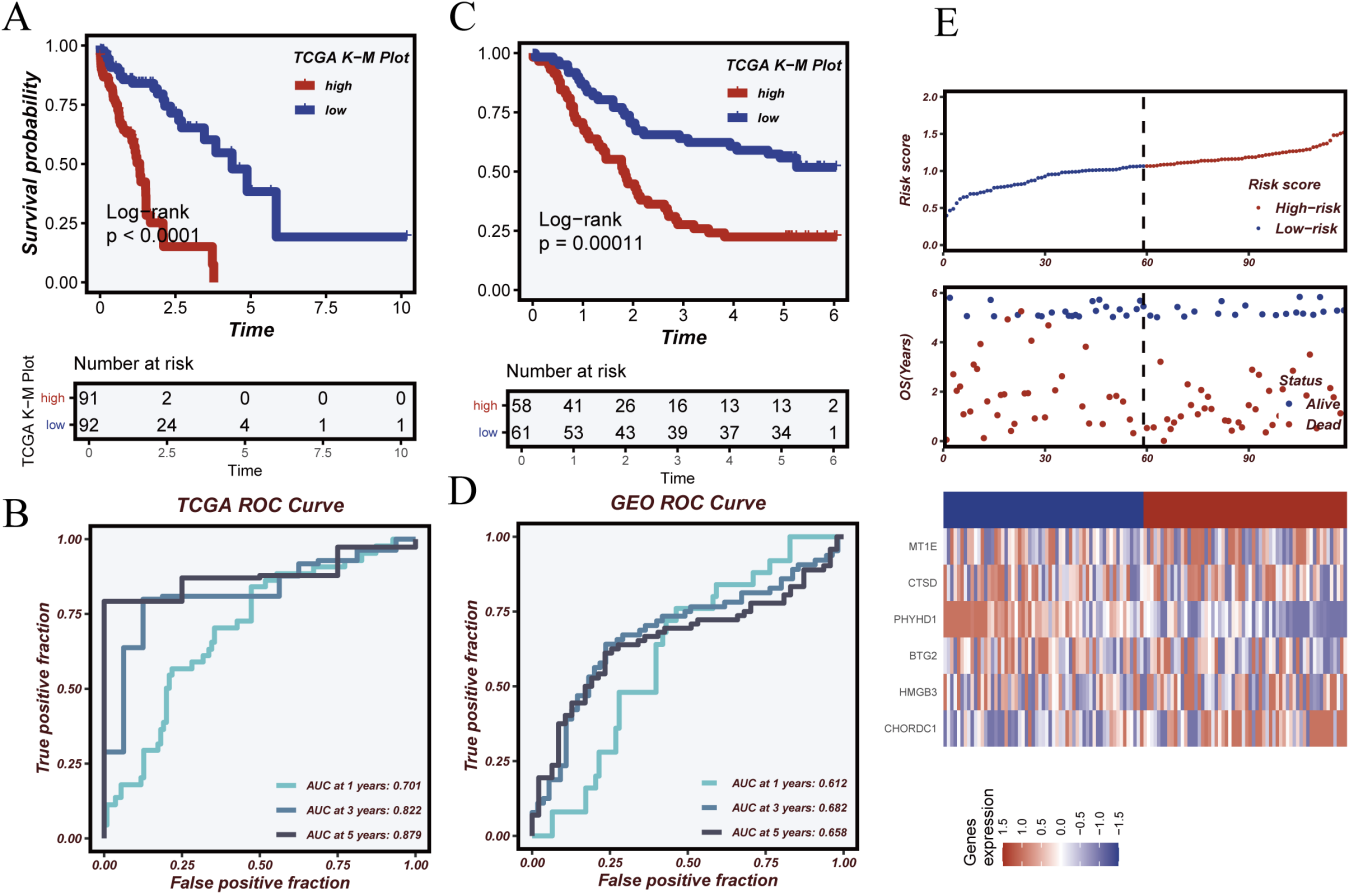
Model validation and prognostic performance evaluation. **(A)** Kaplan–Meier survival analysis in the TCGA cohort showing significantly worse survival in the high-risk group. **(B)** Time-dependent ROC curves in the TCGA cohort assessing model performance at 1, 3, and 5 years. **(C)** Kaplan–Meier survival analysis in the GSE53624 validation cohort. **(D)** Time-dependent ROC analysis in the GSE53624 cohort. **(E)** Distributions of risk score, survival status, and heatmap of key gene expression, illustrating differences between risk groups.

In the TCGA cohort, Kaplan–Meier analysis (**Figure 6A**) revealed significantly poorer survival in the high-risk group (log-rank p < 0.0001). Time-dependent ROC analysis (**Figure 6B**) showed AUCs of 0.812, 0.823, and 0.812 at 1, 3, and 5 years, respectively. In the independent GSE53624 validation cohort, similar trends were observed: high-risk patients had significantly worse survival (**Figure 6C**; log-rank p = 0.00029), and ROC curves (**Figure 6D**) showed AUCs of 0.612, 0.659, and 0.589 at 1, 3, and 5 years. Risk score distributions, survival status, and gene expression patterns (**Figure 6E**) further confirmed the model’s predictive ability. Patients with higher risk scores exhibited shorter survival and distinct gene expression profiles. These results support the robustness and clinical utility of the model for stratifying patients by prognosis.

### 3.8 Independence and clinical applicability of the risk model

**Figure 7:**
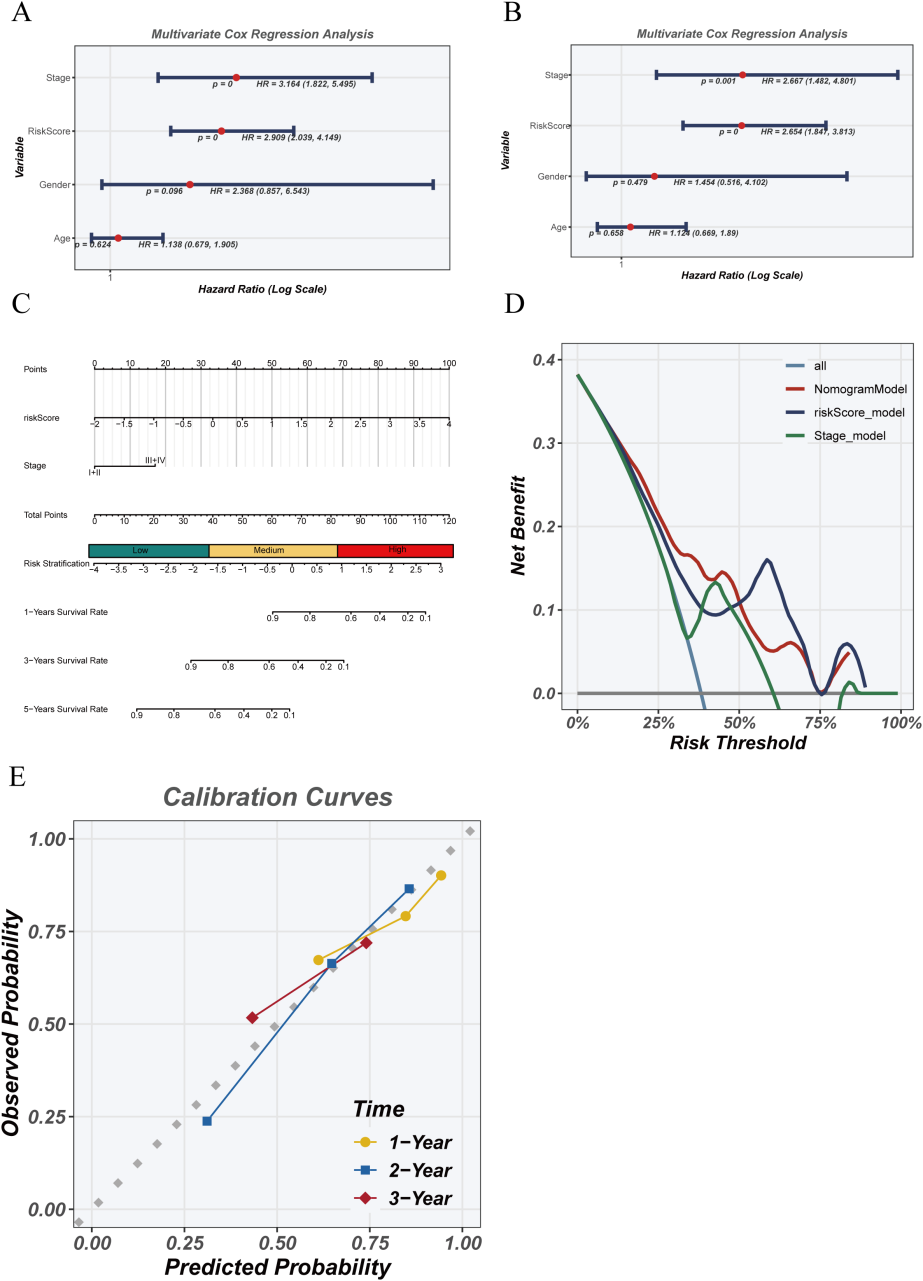
Independence and clinical utility of the risk model. **(A)** Univariate Cox regression analysis of clinical variables and risk score in the TCGA cohort. **(B)** Multivariate Cox regression analysis assessing whether risk score is an independent prognostic factor. **(C)** Nomogram combining risk score and clinical stage to predict 1-, 2-, and 3-year survival probabilities. **(D)** Decision curve analysis (DCA) evaluating the net clinical benefit of risk score, staging, and combined models. **(E)** Calibration curves comparing predicted and observed survival at 1, 2, and 3 years.

Univariate and multivariate Cox regression analyses were conducted to assess the independent prognostic value of the risk score in the TCGA cohort (**Figures 7A, B**). Univariate analysis showed that risk score (HR = 2.09, 95% CI: 1.283–4.448, p < 0.01) and clinical stage (HR = 2.154, 95% CI: 1.822–2.545, p < 0.001) were significantly associated with survival, whereas age and gender were not. Multivariate analysis confirmed that risk score remained an independent prognostic factor (HR = 1.654, 95% CI: 1.154–4.403, p < 0.01) after adjusting for clinical covariates. A nomogram (**Figure 7C**) integrating the risk score and stage was developed to predict 1-, 2-, and 3-year survival probabilities. Decision curve analysis (**Figure 7D**) demonstrated superior net benefit for the risk model and combined model compared to staging alone, particularly at lower risk thresholds. Calibration curves (**Figure 7E**) confirmed excellent concordance between predicted and observed survival, supporting the model’s reliability for personalized prognostic assessment.

### 3.9 Pathway enrichment analysis

**Figure 8:**
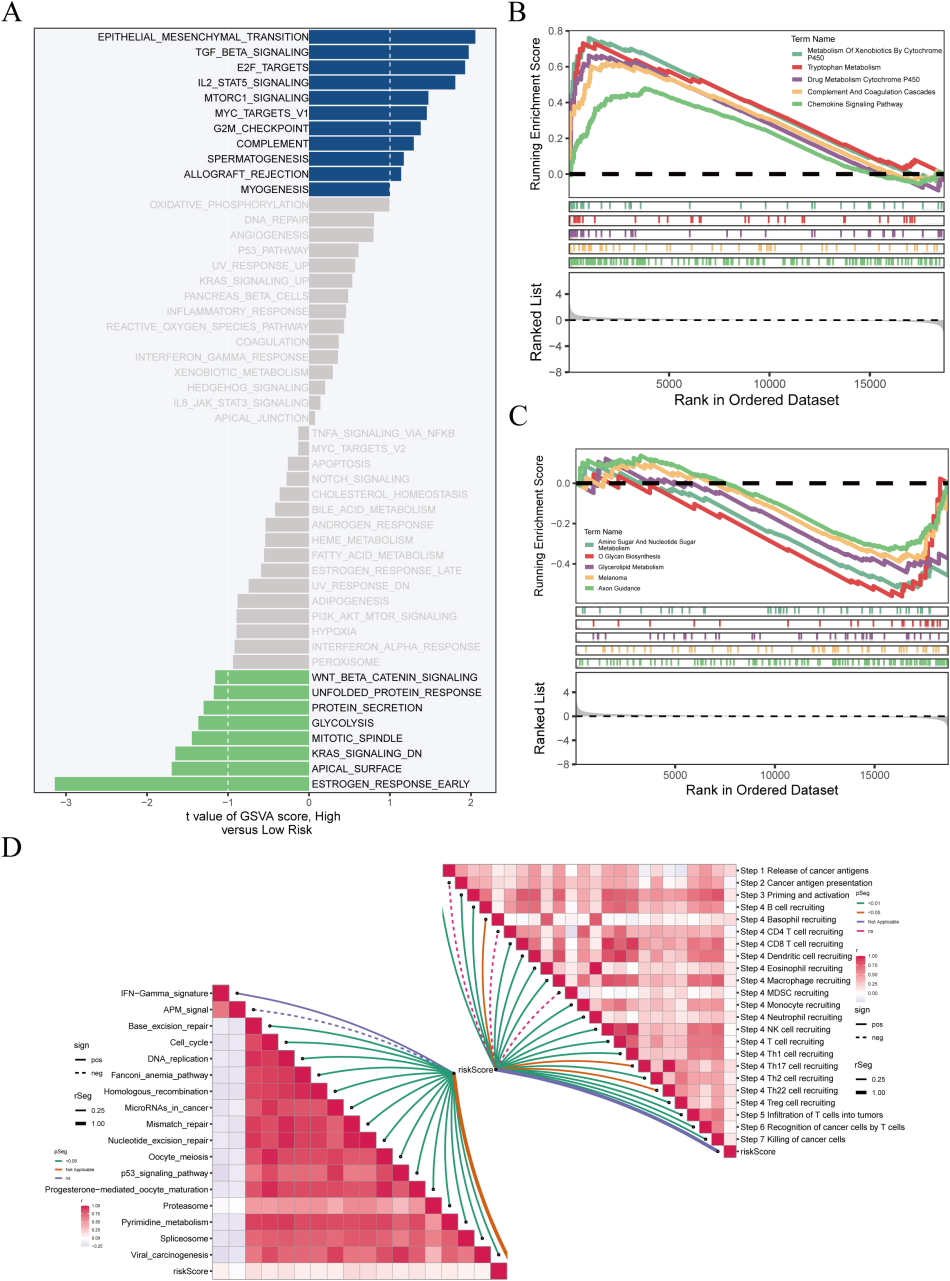
Enrichment analysis reveals biological differences between risk groups. **(A)** GSVA results showing significantly enriched pathways in high-risk (blue) and low-risk (green) groups; x-axis indicates t-value. **(B, C)** GSEA of pathways enriched in high- and low-risk groups, with enrichment score on the y-axis and ranked genes on the x-axis. **(D)** ssGSEA of immune-related pathways; left panel shows correlations between pathways, right panel shows associations with immune infiltration and risk score.

GSVA and GSEA were used to explore pathway differences between high- and low-risk groups. GSVA results (**Figure 8A**) revealed enrichment of TGF-β, IL2–STAT5, G2M checkpoint, MYC targets, and immune-related pathways (e.g., allograft rejection, IFN-γ response) in the high-risk group, suggesting enhanced proliferation and immune dysregulation. Conversely, the low-risk group was enriched for Wnt/β-catenin, lipid metabolism, and DNA repair pathways, indicating a more stable phenotype. GSEA (**Figures 8B, C**) confirmed enrichment of cell cycle and immune suppression–related pathways in the high-risk group, while Glycerolipid metabolism, Axon guidance, and Amino sugar metabolism were prominent in the low-risk group. Although GSVA showed EMT-related pathway activation, EMT was not significantly enriched by GSEA, suggesting that EMT may occur in specific subpopulations rather than the entire high-risk cohort. ssGSEA-based correlation analysis (**Figure 8D**) showed that T cell function, inflammatory response, and cell cycle pathways were more active in the high-risk group, while metabolic pathways were enriched in the low-risk group, highlighting differences in tumor biology and potential therapeutic responses.

### 3.10 Tumor microenvironment characterization and immune infiltration evaluation

**Figure 9:**
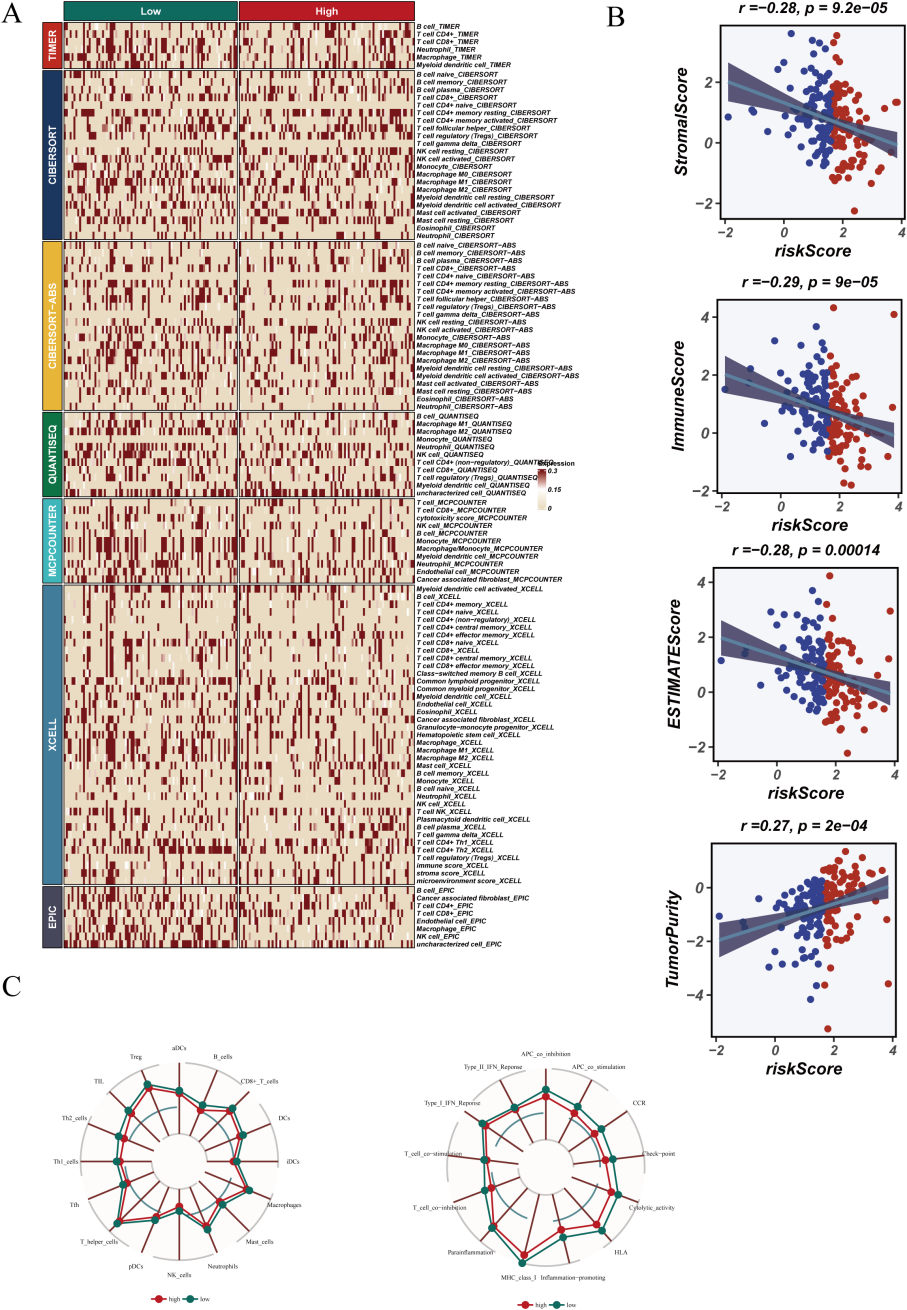
Tumor microenvironment (TME) characteristics and immune infiltration. **(A)** Immune cell infiltration levels across risk groups. **(B)** Correlation analysis using ESTIMATE scores. **(C)** Radar chart showing immune function scores in high- and low-risk groups.

Immune cell infiltration, ESTIMATE scores, and immune functions were compared between risk groups. No significant differences in immune cell composition were found (**Figure 9A**). However, ESTIMATE analysis (**Figure 9B**) showed that StromalScore, ImmuneScore, and ESTIMATEScore were negatively correlated with risk score (StromalScore: r = −0.28, p < 0.05; ImmuneScore: r = −0.29, p < 0.05; ESTIMATEScore: r = −0.28, p < 0.05), whereas TumorPurity was positively correlated (r = 0.27, p = 0.0002), suggesting reduced stromal/immune content and increased tumor cell dominance in the high-risk group.

Radar plots (**Figure 9C**) demonstrated higher immune activity in the low-risk group, supporting the presence of an immunosuppressive microenvironment in high-risk patients, which may contribute to worse outcomes and altered treatment response.

### 3.11 Somatic mutation landscape and model gene mutations

We analyzed mutations in 159 samples to characterize the mutational landscape and model gene alterations. Mutation profiling (**Supplementary Figure 3A**) revealed high mutation frequencies in TTN, MUC16, SYNE1, and TP53, predominantly missense mutations. No significant differences in mutation patterns were observed across clinical subgroups. Summary plots (**Supplementary Figure 3B**) indicated dominant SNV types were C>T and C>A transitions, with variable TMB across patients. Mutation analysis of model genes (**Supplementary Figure 3C**) showed low mutation frequencies, with CHORDC1 and HMGB3 mutated in ~1% of samples and no mutations in BTG2 or CTSD, suggesting their roles may lie more in transcriptional regulation than genetic alterations. Co-mutation analysis (**Supplementary Figure 3D**) revealed significant co-occurrence between TP53 and TTN, while other gene pairs showed no significant associations. These results provide insights into mutation-driven heterogeneity and potential combinatorial mechanisms.

### 3.12 Drug sensitivity prediction

To evaluate treatment responsiveness, drug IC50 values were predicted using the GDSC database. Patients in the low-risk group exhibited significantly increased sensitivity to multiple agents, including AZD1480 (JAK2 inhibitor), SB-505124 (TGF-βR inhibitor), Bortezomib, and Erlotinib (EGFR inhibitor) (**Supplementary Figure 4A**). Targeted agents such as KRAS(G12C) inhibitors, ERK inhibitors, PRIMA-MET, and Osimertinib also showed lower IC50s in low-risk patients, suggesting potential benefit. Conversely, some drugs (**Supplementary Figure 4B**), including ADZ4547 (FGFR inhibitor) and CGP-60474 (CDK inhibitor), had higher IC50s in low-risk patients, indicating possible selective efficacy in the high-risk group. These findings offer valuable guidance for individualized therapy and highlight the potential of risk score–guided treatment selection.

### 3.13 Functional validation of HMGB3

**Figure 10:**
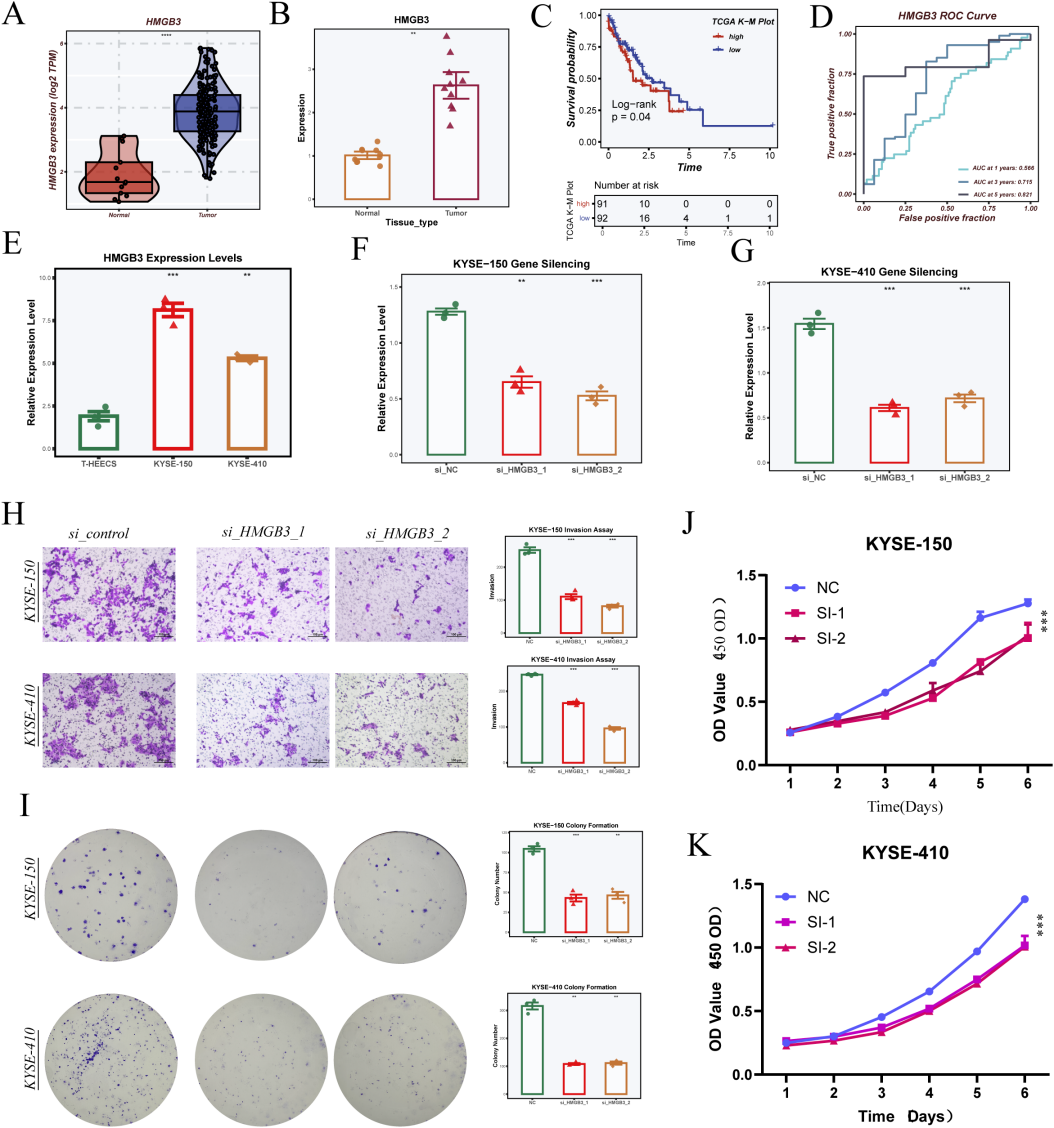
Validation of model gene HMGB3 through cell experiments. **(A)** HMGB3 expression levels in the TCGA-ESCA dataset. **(B)** HMGB3 expression levels in samples from Tianjin Chest Hospital, validated by PCR. **(C)** Survival analysis comparing high and low HMGB3 expression groups. **(D)** ROC curve analysis of HMGB3 gene expression for 1-, 3-, and 5-year survival prediction. **(E)** HMGB3 expression levels in three cell lines (T24, KYSE-150, KYSE-410). **(F, G)** Silencing effects of HMGB3 in KYSE-150 and KYSE-410 cell lines, showing relative expression levels after siRNA treatment. **(H)** Transwell invasion assays in KYSE-150 and KYSE-410 cells with HMGB3 knockdown, with statistical analysis on invasion rate. **(I)** Colony formation assays in KYSE-150 and KYSE-410 cells with HMGB3 silencing, showing colony count comparisons. **(J, K)** CCK-8 assays measuring cell proliferation in KYSE-150 **(J)** and KYSE-410 **(K)** cells after HMGB3 knockdown, with statistical analysis of OD values over 6 days. Data are presented as mean ± SD. *p < 0.05, **p < 0.01, ***p < 0.001, ****P < 0.0001 by Student’s t-test unless otherwise indicated.

We performed PCR to detect the expression levels of six model genes (The primer sequences are shown in **Supplementary Table 1**) in ESCC samples surgically resected from Tianjin Chest Hospital (**Supplementary Figure 5**). Among these six genes, HMGB3 was selected for further validation in cellular experiments. The PCR results indicated that the expression level of HMGB3 was consistent with the bioinformatics analysis (**Figures 10A, B**). Survival analysis showed that patients with high HMGB3 expression had significantly poorer prognosis, suggesting a potential association with adverse outcomes (**Figure 10C**). ROC curve analysis further validated the prognostic potential of HMGB3, demonstrating good performance in predicting 1-, 3-, and 5-year survival (**Figure 10D**).

Experimental results in cell lines revealed that HMGB3 expression was significantly higher in esophageal cancer cells compared to normal esophageal epithelial cells (**Figure 10E**). To further explore the functional role of HMGB3, we silenced HMGB3 expression in two esophageal cancer cell lines using siRNA and confirmed successful knockdown by PCR (**Figures 10F–G**). Transwell invasion assays showed that silencing HMGB3 significantly inhibited the invasive ability of both cell lines (**Figure 10H**). Additionally, colony formation assays demonstrated that silencing HMGB3 suppressed cell proliferation (**Figure 10I**). Finally, CCK-8 assays revealed that HMGB3 knockdown significantly inhibited cell proliferation in both KYSE-150 and KYSE-410 cell lines, with statistically significant differences in OD values over the course of 6 days (**Figures 10J, K**).

## 4 Discussion

In this study, we performed a high-resolution single-cell transcriptomic analysis of esophageal cancer, revealing substantial intratumoral heterogeneity within epithelial cell populations. We identified multiple subpopulations exhibiting putative malignant features and, based on these findings, developed a multi-gene risk score model strongly associated with patient prognosis. Unlike previous models derived from bulk RNA-seq data that rely on differentially expressed genes at the tissue level (36), our study innovatively leveraged single-cell resolution to screen model genes at the cellular subpopulation level. This approach enabled the precise identification of malignant programs hidden within tumor epithelial subsets, which are otherwise masked in bulk data, thereby significantly enhancing the biological specificity and interpretability of the prognostic model.

By applying the inferCNV algorithm, we were able to identify epithelial cell subpopulations with distinct genomic instability features within the single-cell atlas of esophageal cancer. Pseudotime trajectory analysis further reconstructed their potential evolutionary paths, and the distribution of malignant subsets along different branches suggested that these clusters may represent diverse functional states or developmental stages of malignant transformation. Moreover, transcription factor activity inference revealed the subcluster-specific expression of regulators such as FOXC1 and E2F1, highlighting the central role of transcriptional regulatory networks in shaping tumor phenotypes. Notably, FOXC1 has been implicated in the regulation of stemness, EMT, and immune evasion (37–39), while E2F1 is widely known for its involvement in cell cycle activation and DNA replication, serving as a hallmark regulator of proliferative potential in various cancers (40–42). The enrichment of these transcription factors in malignant subclusters underscores their role in driving heterogeneity and promoting aggressive cellular states.

Among the six genes incorporated into our prognostic model, each plays a key role in tumor-related biological processes, reflecting the multi-layered regulation of epithelial cell heterogeneity in ESCC. HMGB3, a member of the high mobility group box family, regulates chromatin remodeling and transcription. It has been reported to be upregulated in several solid tumors and is associated with enhanced proliferation, EMT, and resistance to radiotherapy or chemotherapy (43–45). To further explore the biological relevance of the identified risk signature and validate its functional impact, we performed a series of *in vitro* experiments focusing on key model genes. These functional assays aimed to confirm the role of critical regulators uncovered through computational analysis and to provide mechanistic insights into their contributions to tumor progression. In our study, HMGB3 was significantly upregulated in malignant epithelial subpopulations, and was identified as a high-risk gene in the prognostic model. To further validate its function, we performed a series of *in vitro* experiments. qRT-PCR assays confirmed elevated HMGB3 expression in ESCC tissues compared to adjacent normal tissues. In functional assays, HMGB3 knockdown in ESCC cell lines led to a marked reduction in cell proliferation (CCK-8 assay), invasive capacity (Transwell assay), and colony-forming ability (clonogenic assay). These findings provide direct experimental evidence supporting HMGB3 as a critical driver of ESCC malignancy, and reinforce its potential as a therapeutic target.

CHORDC1, another model gene, encodes a co-chaperone of heat shock protein 90 (HSP90). Although its role in cancer remains underexplored, it has been implicated in stress adaptation via maintaining proteostasis and protein folding, which may contribute to tumor cell survival and immune evasion in the tumor microenvironment (46–48). CTSD, a lysosomal protease, is involved in extracellular matrix degradation and tumor invasion, and its overexpression has been associated with increased aggressiveness in multiple cancer types (49–51). In our analysis, CTSD also emerged as a high-risk gene linked to poor prognosis.

In contrast, BTG2, MT1E, and PHYHD1 were identified as protective factors in our model, potentially functioning as suppressors of tumor progression. BTG2 is a well-known anti-proliferative gene that mediates p53-dependent cell cycle arrest and DNA damage repair. It is frequently downregulated in digestive tract malignancies and may contribute to tumor suppression in ESCC. MT1E, a member of the metallothionein family, regulates oxidative stress responses and metal ion homeostasis, and has been linked to reduced invasiveness and improved immune infiltration (52–54). PHYHD1, though rarely studied in cancer, may be involved in lipid metabolism and redox balance. In our study, its low expression was associated with high-risk scores, suggesting a potential role in metabolic reprogramming and tumor microenvironment adaptation (55–57).

Collectively, the six-gene risk model integrates diverse mechanisms of tumor promotion and suppression and exhibits strong prognostic value. Beyond survival prediction, it reflects the molecular underpinnings of chromatin remodeling, proteostasis, extracellular matrix interaction, immune evasion, oxidative stress resistance, and metabolic adaptation—key hallmarks of cancer biology.

Importantly, our model demonstrated robust predictive performance in two independent cohorts and remained an independent prognostic factor in multivariate Cox regression analysis. Moreover, it effectively stratified patients based on the immune contexture. High-risk patients displayed an immunosuppressive tumor microenvironment characterized by reduced stromal and immune cell infiltration, increased tumor purity, and activation of immunosuppressive pathways, in line with their unfavorable prognosis. Given the strong association between the risk model and the immune microenvironment observed in our study, it is important to recognize that TME plays a central role in shaping immunotherapy outcomes. The TME consists of immune cells, stromal components, cytokines, and tumor-associated factors, whose dynamic interactions can either promote antitumor immunity or facilitate immune escape. Recent evidence suggests that functional states and spatial distribution of T cells, tumor-associated macrophages, and cancer-associated fibroblasts critically influence immune responses to therapy (58). Furthermore, cytokines such as TGF-β and IL-10 modulate immune cell recruitment and polarization, contributing to treatment resistance (33). These findings underscore the importance of TME heterogeneity in interpreting risk stratification and predicting immunotherapy efficacy in esophageal cancer.

These features were also accompanied by differential enrichment of immune signaling, cell cycle progression, and interferon response pathways, suggesting complex interactions between tumor cells and the immune milieu. Compared to conventional models built from bulk datasets such as TCGA or GEO, our single-cell-based approach provides a biologically grounded foundation for prognostic modeling, capturing the intrinsic malignant programs at the cellular origin. This enhances model interpretability and offers a framework for linking specific cell subpopulations to clinical outcomes and immune responsiveness.

Notably, the majority of model genes had low mutation frequencies, suggesting that their oncogenic effects are likely driven by transcriptional dysregulation or epigenetic alterations rather than direct genomic mutations. This highlights the importance of integrating expression-level features rather than relying solely on mutational data when modeling tumor behavior. Drug sensitivity analysis further showed that the risk model could predict differential responses to chemotherapy and targeted therapies, indicating its potential clinical utility in guiding individualized treatment decisions.

Despite these strengths, our study has some limitations. The model requires validation in large, prospective, multi-center cohorts. Additionally, while we confirmed the functional role of HMGB3 through *in vitro* experiments, further mechanistic studies at the protein level and *in vivo* models are necessary to clarify the downstream pathways involved. Future work will include subcutaneous xenograft assays to evaluate tumorigenic capacity and lung metastasis models to assess invasive potential. Furthermore, pathway-specific *in vivo* experiments, such as modulation of the HIPPO and EMT pathways, will be conducted to validate the regulatory networks identified. Moreover, the roles of other model genes such as CHORDC1 and PHYHD1 warrant further experimental exploration, particularly in the context of tumor metabolism and immune escape.

In conclusion, our study provides a single-cell–based framework for dissecting epithelial cell heterogeneity in ESCC and demonstrates how integrating scRNA-seq with functional and clinical data can yield robust prognostic models. The six-gene risk score model not only predicts patient outcomes but also reflects key biological processes such as chromatin remodeling, cell proliferation, immune modulation, oxidative stress response, and metabolic reprogramming. These findings offer new insights into the molecular landscape of esophageal cancer and provide a valuable foundation for future efforts in precision oncology and therapeutic stratification.

## Data Availability

The original contributions presented in the study are included in the article/**Supplementary Material**. Further inquiries can be directed to the corresponding author.
