# Supplementary material for: Malignant epithelial cell marker–driven risk signature enables precise stratification in esophageal cancer
Source: Front Immunol. 2025 May 27;16:1610991. doi: 10.3389/fimmu.2025.1610991 (PMC12149094; doi:10.3389/fimmu.2025.1610991)
Supplement: Supplementary file 1 [file DataSheet1.docx]

Supplementary Material

## Supplementary Figures


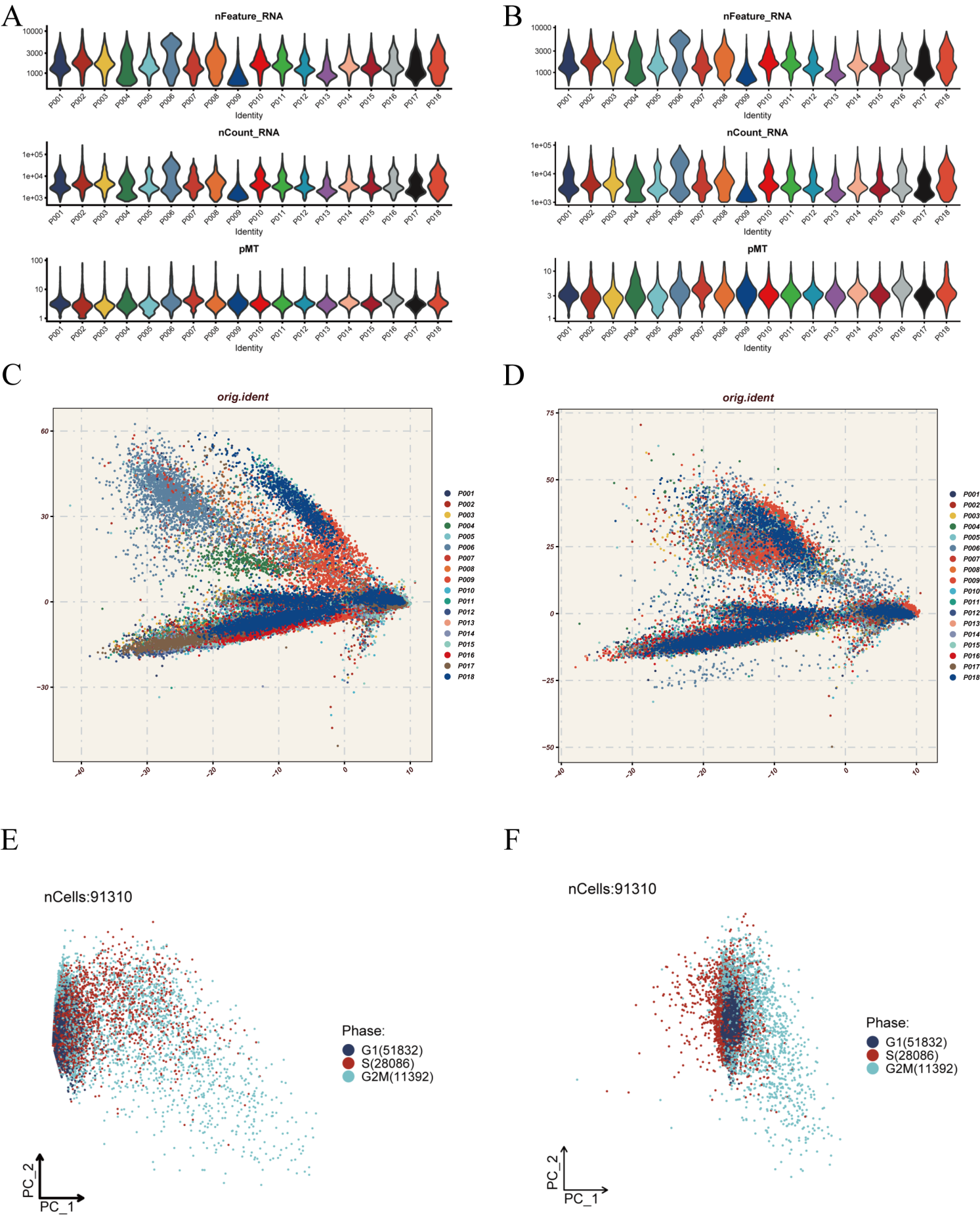


**Supplementary Figure 1: Quality control and batch effect correction.** (A–B) Distributions of nFeature_RNA, nCount_RNA, and mitochondrial gene percentage (pMT) before and after quality control, visualized as violin plots. (C–D) Sample distributions in reduced dimensional space before and after batch correction using the Harmony algorithm. (E–F) PCA visualization of cells by cell cycle phase (G1, S, G2M) before and after cell cycle correction using ScaleData() on S.Score and G2M.Score.


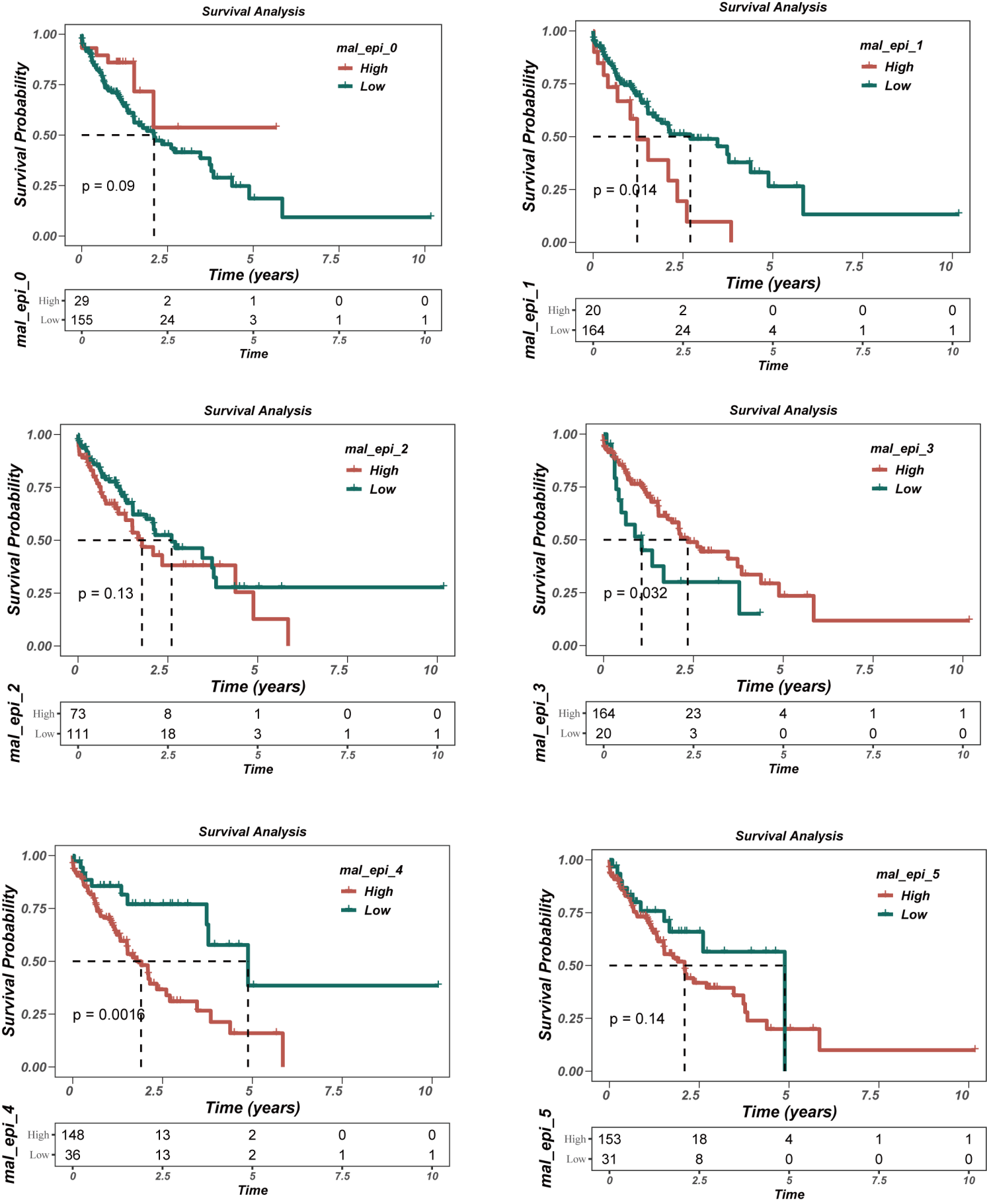


**Supplementary Figure 2: Prognostic analysis of epithelial subpopulations.** Kaplan–Meier survival curves comparing high vs. low expression groups for each malignant epithelial subcluster (Cluster 0–5), showing differences in overall survival.


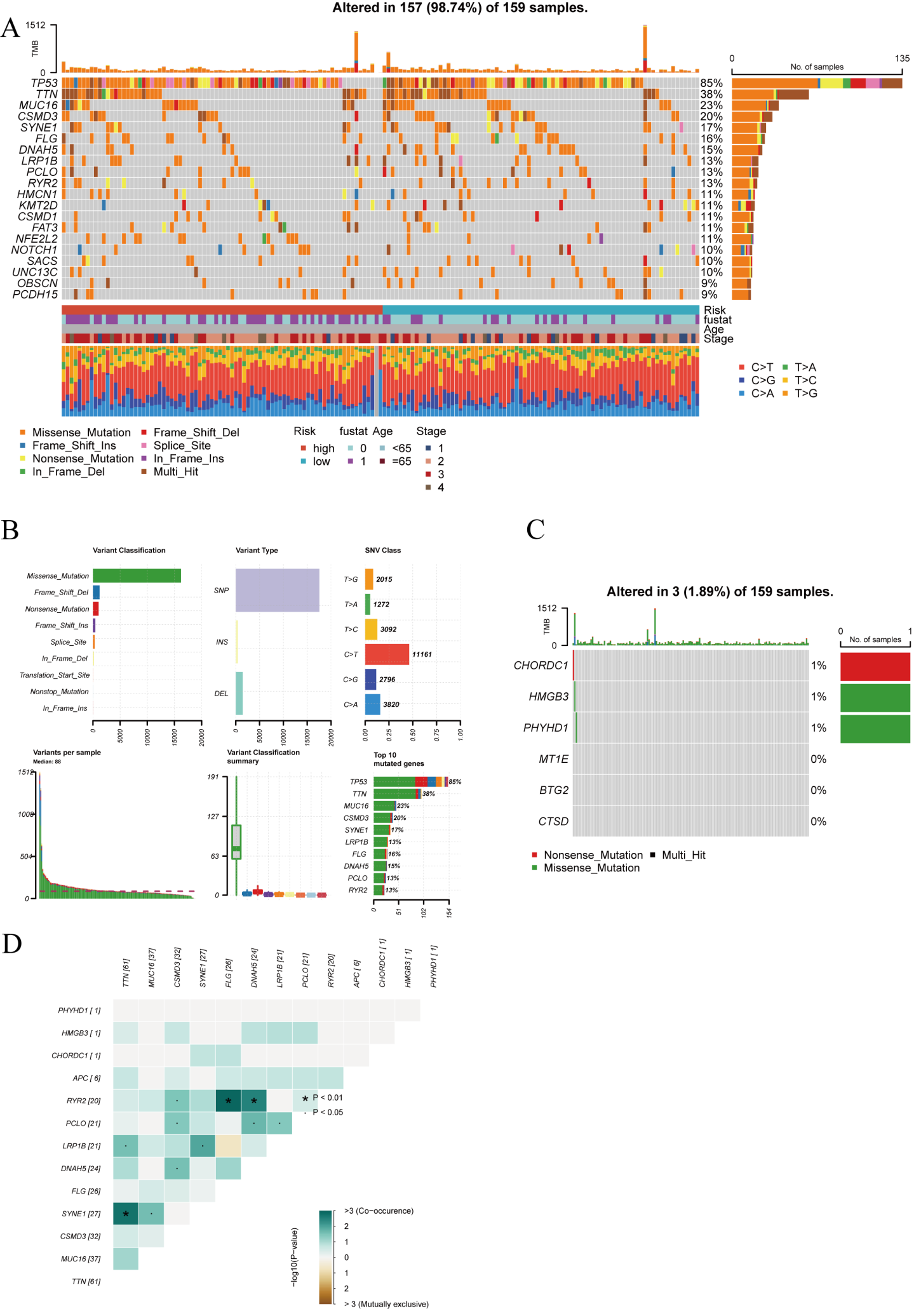


**Supplementary Figure 3: Mutation landscape and model gene mutation analysis.** (A) Oncoplot showing top 20 frequently mutated genes in 159 samples, with annotations of clinical features (risk group, age, stage). (B) Overview of mutation types, SNV categories, transition/transversion ratio, and tumor mutational burden (TMB). (C) Mutation status of model genes. (D) Co-mutation and mutual exclusivity analysis among frequently mutated genes, with p-values indicating statistical significance.


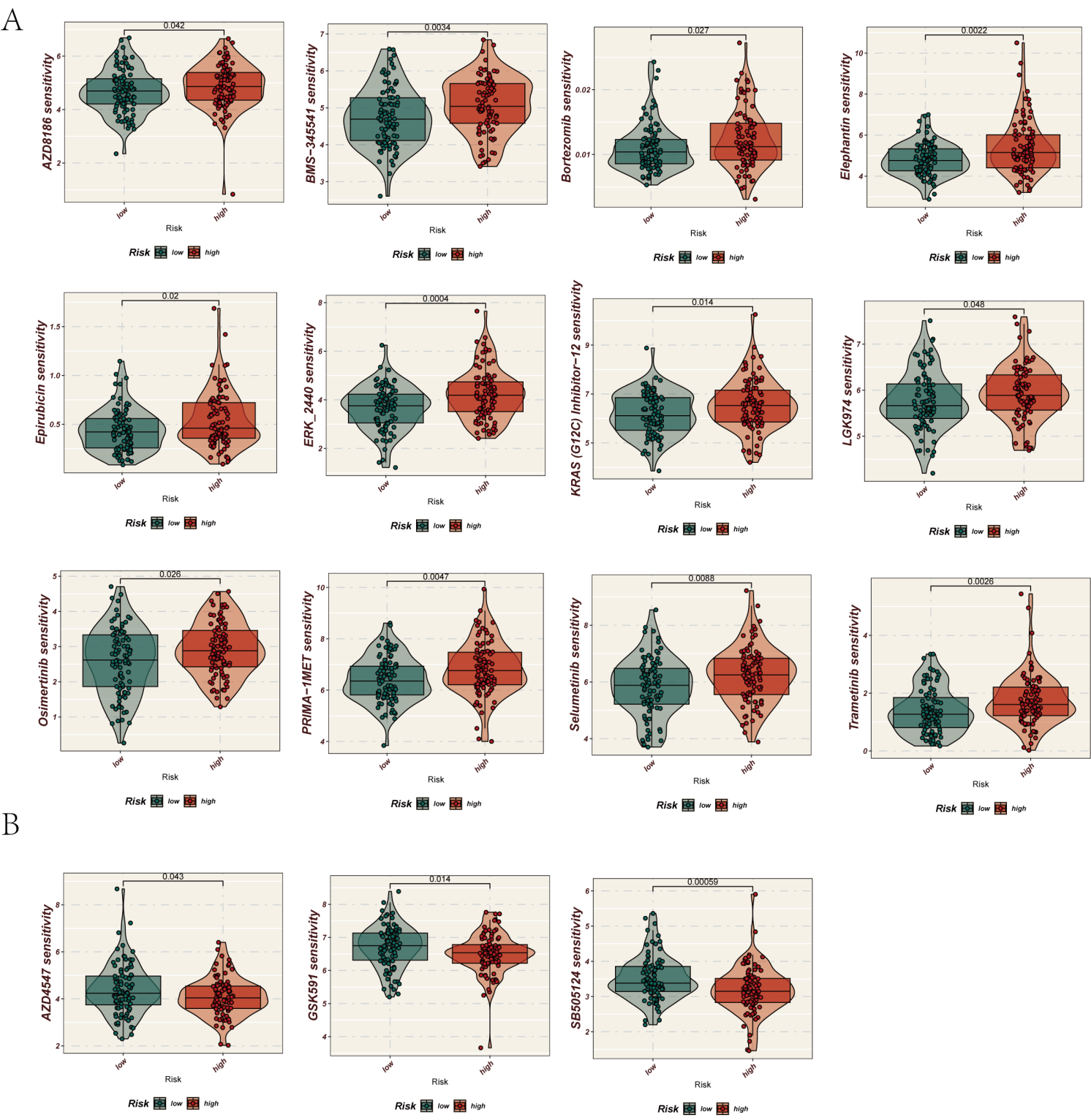


**Supplementary Figure 4: Drug sensitivity analysis.** (A) Lower predicted IC50 values for several drugs in the low-risk group, indicating higher sensitivity. (B) Higher IC50 values for certain drugs in the low-risk group, suggesting potential resistance.


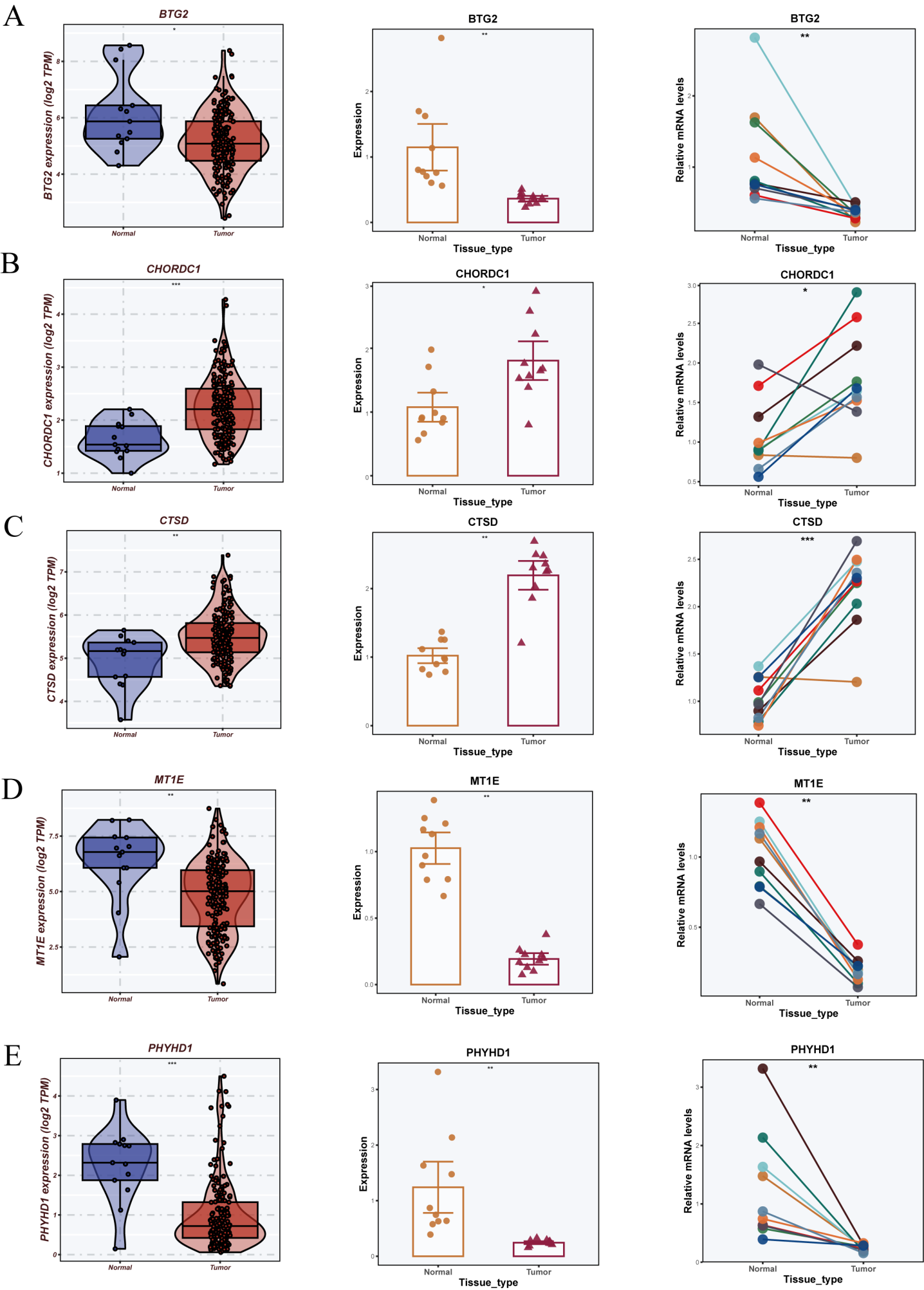


**Supplementary Figure 5: Validation of model gene expression levels.** (A) BTG2, (B) CHORDC1, (C) CTSD, (D) MT1E, and (E) PHYHD1 expression levels in the TCGA-ESCA dataset (left) and PCR validation of expression levels in surgical samples from Tianjin Chest Hospital (right). The right panel shows results for paired and unpaired samples, with expression level differences marked for statistical significance.

| Gene |  | **Sequence (5'->3')** |
| --- | --- | --- |
| HMGB3 | **Forward primer** | GCCGGGAAGGAAGAAGCAAT |
|  | **Reverse primer** | TTCCCGGACATCGTCTTCCA |
| CHORDC1 | **Forward primer** | CCTGTGACGGTGGGAAAAGA |
|  | **Reverse primer** | GTATGTGCAAGCATCGTCGG |
| CTSD | **Forward primer** | GCTGATTCAGGGCGAGTACA |
|  | **Reverse primer** | CTCTGGGGACAGCTTGTAGC |
| BTG2 | **Forward primer** | GAGGCACTCACAGAGCACTA |
|  | **Reverse primer** | TGGGGTCCATCTTGTGGTTG |
| MT1E | **Forward primer** | CGCCACTGGTGGCTCC |
|  | **Reverse primer** | TGAGTTCCCTCCCAACCTGA |
| PHYHD1 | **Forward primer** | CCCTCCGGAGAAATCCATCA |
|  | **Reverse primer** | GCCAAAGTGAGGTTGCTTAAAGA |
| Si-HMGB3-1 | **Forward primer** | GGAAGUGAUCAUCUCCGAUTT |
|  | **Reverse primer** | AUCGGAGAUGAUCACUUCCTT |
| Si-HMGB3-2 | **Forward primer** | GGUCUUCGCCUUGAUUCAUTT |
|  | **Reverse primer** | AUGAUAAAGGCGAAGACCTT |

**Supplement Table1:The primer sequences of model genes and siRNA sequences.**
